# Selected Texan K-12 Educators’ Perceptions of Youth Suicide Prevention Training

**DOI:** 10.3390/ijerph191912625

**Published:** 2022-10-03

**Authors:** Melanie McKoin Owens, Alexis Zickafoose, Gary Wingenbach, Sana Haddad, Jamie Freeny, Josephine Engels

**Affiliations:** 1 Department of Agricultural Leadership, Education and Communications, Texas A&M University, College Station, TX 77843, USA; 2 Mental Health America of Greater Houston, Houston, TX 77098, USA

**Keywords:** youth suicide prevention, training, educators, perceptions

## Abstract

K-12 school personnel may be frontline responders for youth contemplating suicide or other harmful behaviors. Therefore, the purpose of this preliminary study was to determine selected K-12 educators’ perceptions of youth suicide prevention (YSP) training. A longitudinal trend survey with repeated measures and proportionally stratified random samples of K-12 personnel from nine Texas independent school districts provided data. Participants’ perceived knowledge of the YSP content showed significant appreciative gains between pre- and follow-up post-tests. Likewise, their confidence levels for helping students at risk of suicide and approaching other adults to talk about students at risk of suicide rose significantly between pretests and follow-up post-tests. This preliminary study reinforces the value of training educators to acquire content knowledge and confidence boosting opportunities for engagement in difficult dialogue about suicidality. YSP training helped improve educators’ confidence to engage with others about students’ mental health concerns, calling attention to the importance of identifying early warning signs that may aid in early support and prevention of youth mental health concerns.

## 1. Introduction

Suicide was the second leading cause of death in the United States among 10–35-year-olds in 2019 [1]. Adolescent suicidality rates are on the rise [2,3]. Suicidality includes ideation, attempts, and completed suicides [4]. Researchers found more than 16% of adolescents are diagnosed with mental health illnesses including suicidality; of those adolescents about 49% go untreated [5]. Lack of care and under-reporting suicidality hinders understanding the extent of youth’s mental health concerns. Youth are influenced by family, community constituents, school administrators, and other individuals or mediums, whether helpful or harmful [6]. Educators’ daily contact with students fosters an environment whereby school personnel might become frontline responders for students who need help with mental health concerns [6]. Therefore, educators should seize upon opportunities to promote student development and wellbeing [7].

### 1.1. Signs and Symptoms of Youth Suicidality

Destruction from hurricanes and school shootings are two examples of traumatic events that may negatively affect youth’s mental health. Situations causing trauma may produce heightened concerns of suicidality threats [2,8]. Research shows mental health concerns initiate during adolescence, potentially when youth are in K-12 settings [9]. Emotional and physical abuse, psychological diagnoses, acceptance and relatability with peers, family, or school administrators, self-acceptance and understanding, and cognitive and emotional competence are among the factors that positively or negatively impact youth suicidality [2,10,11]. Promoting positive mental health development alleviates some factors contributing to negative mental health issues [12,13]. Environments promoting engagement and discussion about mental health concerns may discourage thoughts of suicide or other harmful behaviors [14]. Past events demonstrate that some youth contemplating suicide or other harmful behaviors [15,16] are particularly vulnerable after school shootings. There exists an urgency to provide youth suicide prevention training programs in all schools.

### 1.2. Youth Suicide Prevention Training Programs

Researchers suggest mental health support and awareness programs are necessary for healthy societies [12,17,18,19]. Care for youth’s mental health concerns extend beyond specialized mental healthcare providers [20]. Educators and schools provide communities of care for students. School personnel may see students with emotional distress; thus, school staff are often expected, regardless of training, to serve as frontline pseudo-mental health professionals [6,14,21]. Equipping K-12 staff with knowledge of the signs and symptoms of youth suicide or other harmful behaviors and the actions to prevent them creates significant changes in education [21]. School administrators identified gaps in teachers’ preparedness for addressing youth suicidality [22,23,24]. Youth mental health awareness and training was limited to theoretical concepts rather than practical under-standing and application for staff [20]. Therefore, interventions that include awareness of the effects of trauma, youth suicide and other harmful behaviors, and the actions to take in helping at-risk youth are critical to increasing school staffs’ preparedness in youth suicide prevention [25].

Programs such as Mental Health First Aid and Youth Mental Health First Aid include curricula tailored to meet educators’ mental health training needs [26]. Mental Health First Aid training provides tools for identifying individuals navigating mental health crises to triage care appropriately [27,28]. Educators who recognize and prevent youth suicide or other harmful behaviors are crucial in maintaining safe and caring schools. However, effective training programs in youth suicide prevention are dependent upon knowing school staffs’ knowledge and perceptions of such topics. Mental Health America of Greater Houston (MHAGH) initiated the Emotional Backpack Program (EBP) in selected Texas school districts to help educators address youth trauma from events such as Hurricane Harvey. The EBP includes multiple training interventions, including Youth Suicide Prevention (YSP), which was the focus of this preliminary study.

Assessing K-12 school staffs’ knowledge and perceptions of YSP training helps MHAGH refine and deliver improved training that strengthens educators’ capacities for addressing youth’s suicidal ideation and other harmful behaviors. Therefore, the purpose of this preliminary study was to determine selected K-12 educators’ perceptions of YSP training. Research objectives were to: (1) test perceived knowledge of the signs/symptoms of students at risk of suicide and the actions to take when a student is at risk of suicide; (2) assess confidence in helping students at risk of suicide; and (3) evaluate confidence in approaching other adults about students at risk of suicide.

## 2. Materials and Methods

This preliminary study was conducted as a part of MHAGH’s Center for School Behavioral Health EBP, which included similar materials and methods reported in [29]’s study. All pertinent YSP methods and materials are reported in following.

### 2.1. Study Design and Procedure

We used a cross-sectional survey design [30] with repeated tests (i.e., surveys) for data collection. The population was all school personnel (*N* ≈ 29,900) from nine Texas independent school districts (Alief, Alvin, Clear Creek, Dickinson, Fort Bend, Goose Creek, Katy, La Porte, and Spring). These school districts experienced significant natural (Hurricane Harvey) and human-made (Santa Fe, Texas school shootings) traumatic events before this preliminary study was completed.

MHAGH notified selected school districts about its YSP training program about 1–2 months in advance of the day (typically in mid-spring) to coincide with school districts’ end-of-year continuing education programs. An online consent script informed each participant that anonymous responses would be used in group format for technical reports and publications; consent required acknowledgement (I Agree) before entry to the training online surveys. Immediately after each YSP training, we used the single-assessment retrospective pre-post design (SARPPD) to administer training session tests. We selected the SARPPD because it freed up time and money needed during YSP training, allowed training staff more time to build rapport with participants, and because the SARPPD is especially useful in conducting surveys that measure participants’ attitudes and beliefs. We noted that post-program SARPPD is not without its own concerns, chief among them are participants’ imperfect memory recall, social desirability, acquiescence, and cognitive dissonance, which may lead to overstated evaluations of program effectiveness [31]. We chose the SARPPD measurement design because we feared response shift bias was more likely with a classic pre/post design and because we believed participants’ ratings of the YSP content would not be affected negatively by the limitations of the SARPPD. The SARPPD afforded time- and cost-efficiencies that were not possible with traditional pre/post designs. Not all participants answered all items or completed all tests; therefore, only group response sets (e.g., group pretest vs. group post-test) were analyzed and reported. Groups differed in number and characteristics; hence, these results are confined to YSP training participants only and should not be inferred to other groups.

MHAGH provided more than 30 YSP sessions in Texas school districts from 2019 to 2020. MHAGH’s training sessions were delivered in-person before the novel Coronavirus 2019 lockdown and virtual workshops began after March 2020. Three MHAGH mental health experts used a train-the-trainer approach to build school staff capacities for addressing students’ behavioral and mental health development. Training sessions included experiential activities such as role play simulation, problem solving scenarios, and small group discussion about youth mental health issues. YSP training topics included recognizing warning signs of suicide, students’ at-risk behaviors, crisis management, and providing prevention or intervention support. Each training session was about 90 min, facilitated by mental health experts or school personnel (peer) trainers, and included at least two school staff per campus. YSP training fulfilled Texas Education Agency requirements for school staff professional development in youth mental health and suicide prevention [32].

### 2.2. Participants

Proportionally stratified (by test type) random samples were drawn from the accessible population (*n* = 1426) of YSP training attendees. Sample sizes were determined using [33]’s methods for deriving probability samples. We calculated random samples using a conservative 50/50 split with a 5% sampling error and a 95% confidence level [33]. These parameters show a sample of 303 represents the population of 1426; we rounded up to 400 to offset mortality threats to internal validity [34].

More than 1400 school personnel attended MHAGH’s YSP training sessions in 2019 and 2020. Of those participants, 400 were randomly selected for data analysis. Note that not all participants answered all questions, such as demographic data, therefore missing data may represent bias in the response set. Most respondents (who did provide demographic data) were White, female, teachers between the ages of 26 and 45 (*M* = 41.73, *SD* = 11.41 (Table 1).

Informed consent was obtained from all subjects and/or their legal guardian(s) by acknowledging agreement with an online consent script before accessing online surveys (i.e., tests). The Texas A&M University Institutional Review Board waived the process to document consent because this study did not involve more than minimal risk. This study was exempt by an Institutional Review Board at Texas A&M University. It is a program evaluation. The YSP training activities and data reported herein are not considered research involving human subjects as defined by DHHS and/or FDA regulations.

### 2.3. Measurement

The YSP intervention included repeated tests (i.e., pre/post-test, 3-month, and year-end tests) of participants’ perceived knowledge of YSP content and confidence in taking actions based on [35]’s program evaluation. All items were measured with retrospective post-then-pre statements using 5-point Likert-type response scales (poor, fair, good, very good, excellent). For example, after participating in the initial YSP intervention, participants were presented with a post-test that included two questions about their perceived knowledge of YSP content. Those questions were stated as: “Before this workshop, my knowledge of the actions to take when a student is at risk of suicide was: (poor…excellent)” and “After this workshop, my knowledge of the actions to take when a student is at risk of suicide was: (poor…excellent)”. About three months later, participants were invited to a follow-up survey, using the same structured questions as in the post-then-pre survey. Minor wording changes resulted in these statements: Before this workshop, my knowledge of the actions to take when a student is at risk of suicide was (poor…excellent); and 3 months after this workshop, my knowledge of the actions to take when a student is at risk of suicide is (poor…excellent). Again, about one year later, participants were asked to provide input in a follow-up survey. Statements included: “Before this workshop, my knowledge of the actions to take when a student is at risk of suicide was (poor…excellent)” and One year after this workshop, my knowledge of the actions to take when a student is at risk of suicide is (poor…excellent). We used the same question structure for other questions in all surveys. We hypothesized (*H*_1_) participants’ perceived knowledge of YSP content would increase after the intervention training.

Confidence in helping students at risk of suicide was measured with participants’ ratings (5-point scale: poor…excellent) of three statements: confidence in approaching a student at risk of suicide, helping at-risk students, and asking a student about suicide. We hypothesized (*H*_2_) that participants’ confidence would increase after training. Confidence in approaching other adults about students at risk of suicide was measured with participants’ ratings (5-point scale: poor…excellent) of three statements: confidence in approaching a school counselor, administrator, and parent or guardian. We hypothesized (*H*_3_) that participants’ confidence in approaching other adults about students at risk of suicide would increase after the intervention.

### 2.4. Statistical Analysis

We assessed normality using Kolmogorov–Smirnov and Shapiro–Wilk test to determine if data were normally distributed. Kolmogorov–Smirnov and Shapiro–Wilk tests showed that data were not normally distributed (Table 2). Based on these outcomes, we used non-parametric tests (Independent-Samples Kruskal–Wallis Test; *k* > 2) to determine if changes occurred in participants’ perceptions of the YSP training content. In cases where the Kruskal–Wallis test revealed significant differences (if the adjusted *p*-value was <0.05), additional analysis were conducted to evaluate pairwise comparisons among groups, while controlling for Type I error across tests with the Bonferroni correction.

## 3. Results

### 3.1. Objective 1

Participants’ perceived knowledge of the YSP content ranged from good (*M*s = 3.22–3.25) in pretests to very good (*M*s = 3.52–3.98) in post- and follow-up tests (Table 3). The Independent-Samples Kruskal–Wallis Test was used to evaluate differences in mean ranks of participants’ perceived knowledge of YSP content increasing after the YSP intervention training (*H*_1_). Significant (*p* < 0.05) differences existed in the mean ranks of knowledge of actions to take, *χ*^2^(3) = 34.20, *p* < 0.001; and knowledge of the signs and symptoms of suicide risk in students, *χ*^2^(3) = 40.28, *p* < 0.001 when analyzed by test. *H*_1_ was fully supported. Levene’s test for equality of variances was violated across groups, *F*(3) = 3.73, *p* = 0.01, for knowledge of actions to take when a student is at risk of suicide, and for knowledge of the signs and symptoms of suicide risk in students *F*(3) = 3.75, *p* = 0.01. Follow-up analysis, controlling for Type I error across tests using the Bonferroni approach, produced an expected outcome, in that participants reported significantly higher levels of agreement with knowledge of actions to take when a student is at risk of suicide in post-, *z* = −4.68, *p* = 0.000, and year-end tests *z* = −4.79, *p* = 0.000 than in pretests. Pretest produced an average rank of 167.13, while post-test had an average rank of 228.73 and year-end test had an average rank of 246.35. Likewise, we found an expected outcome in that participants reported significantly higher levels of agreement with knowledge of the signs and symptoms of suicide risk in students in post-, *z* = −4.92, *p* = 0.000, and year-end tests, *z* = −5.35, *p* = 0.000 than in pretests. Significant differences occurred also between 3-month and year-end tests, *z* = −2.78, *p* = 0.033. Pretest mean ranks produced an average rank of 164.41, post-test had an average rank of 229.01, 3-month had an average rank of 194.31, and year-end test had an average rank of 252.75.

### 3.2. Objective 2

Participants’ confidence in helping students at risk of suicide ranged from good (*M*s = 2.80–3.02) in pretests to good or very good (*M*s = 3.38–4.02) in post- and follow-up tests (Table 4). The Independent-Samples Kruskal–Wallis Test was used to determine if participants’ confidence in helping students at risk of suicide increased after YSP intervention training (*H*_2_). Significant (*p* < 0.05) differences existed in the mean ranks of confidence in approaching a student at risk of suicide, *χ*^2^(3) = 56.33, *p* < 0.001; confidence in helping a student at risk of suicide, *χ*^2^(3) = 42.60, *p* < 0.001; and confidence in asking a student about suicide, *χ*^2^(3) = 47.84, *p* < 0.001 when analyzed by test. *H*_2_ was fully supported. Levene’s test for equality of variances was violated across samples for items, confidence in approaching a student at risk of suicide, *F*(3) = 4.77, *p* = 0.03; confidence in helping a student at risk of suicide, *F*(3) = 4.79, *p* = 0.03; and confidence in asking a student about suicide, *F*(3) = 3.71, *p* = 0.12. Follow-up analysis, controlling for Type I error across tests using the Bonferroni approach, produced expected outcomes, in that participants had significantly more agreement with confidence in helping students at risk of suicide in post-, *z* = −5.28, *p* = 0.000, and year-end tests *z* = −6.69, *p* = 0.000 than they did in pretests. Pretest had an average rank of 157.05, while post-test had an average rank of 227.06 and year-end test 268.59. The year-end test differed significantly from the 3-month test as well, *z* = −2.72, *p* = 0.039. The 3-month average rank was 210.61. Follow-up tests, using the Bonferroni approach, produced another expected outcome; participants reported significantly more confidence in helping a student at risk of suicide after the YSP training in post-test, *z* = −4.24, *p* = 0.000, and year-end tests *z* = −6.05, *p* = 0.000 than they did in pretests. Pretest had an average rank of 162.89, post-test had an average rank of 218.89 and year-end test 263.22. The year-end test differed significantly from the 3-month test as well, *z* = −2.88, *p* = 0.024. The 3-month average rank was 202.08. Finally, our follow-up tests revealed participants’ confidence in asking a student about suicide was significantly greater in post, *z* = −5.19, *p* = 0.000, 3-month, *z* = −2.98, *p* = 0.017, and year-end tests, *z* = −5.91, *p* = 0.000, than it was in the pretest. Pretest had an average rank of 158.71, post-test had an average rank of 228.00, 3-month average rank was 211.97, and year-end test had an average rank of 257.77 (Table 4).

### 3.3. Objective 3

Participants’ confidence in approaching other adults about students at risk of suicide ranged from good (*M*s = 2.65–3.34) in pretests to good/very good (*M*s = 3.42–4.34) in post- and follow-up tests (Table 5). The Independent-Samples Kruskal–Wallis Test was used to determine if participants’ confidence in approaching other adults about students at risk of suicide increased after YSP intervention training (*H*_3_). Significant (*p* < 0.05) differences existed in the mean ranks of confidence in approaching a school counselor about a student at risk of suicide, *χ*^2^(3) = 40.26, *p* < 0.001; confidence in approaching an administrator about a student at risk of suicide, *χ*^2^(3) = 29.72, *p* < 0.001; and confidence in approaching a parent or guardian about a student at risk of suicide, *χ*^2^(3) = 49.92, *p* < 0.001 when analyzed by test. *H*_3_ was fully supported. Levene’s test for equality of variances was violated across samples for items, confidence in approaching a school counselor about a student at risk of suicide, *F*(3) = 6.12, *p* < 0.001; and confidence in approaching an administrator about a student at risk of suicide, *F*(3) = 4.61, *p* = 0.003. Levene’s test for equality of variances was not violated across samples for the item confidence in approaching a parent or guardian about a student at risk of suicide, *F*(3) = 1.69, *p* = 0.17. Follow-up analysis, controlling for Type I error across tests using the Bonferroni approach, produced expected outcomes; participants agreed significantly more with being confident in approaching a school counselor about a student at risk of suicide in post-, *z* = −4.32, *p* = 0.000, 3-month, *z* = −4.54, *p* = 0.002, and year-end tests *z* = −5.97, *p* = 0.000 than in the pretest. Pretest had an average rank of 121.48, while post-test was 174.40, 3-month average rank was 176.77, and year-end had an average rank of 209.75. Follow-up tests, using the Bonferroni approach, produced another expected outcome; participants were significantly more confidence in approaching an administrator about a student at risk of suicide after the YSP training in post-, *z* = −2.99, *p* = 0.017, 3-month, *z* = −3.70, *p* = 0.001, and year-end tests *z* = −6.05, *p* = 0.000 than in pretests. Pretest had an average rank of 127.70, post-test was 172.97, 3-month was 174.3, and year-end test had an average rank of 203.78. Finally, the follow-up tests for participants’ confidence in approaching a parent or guardian about a student at risk of suicide was significantly greater in post, *z* = −5.38, *p* = 0.000, 3-month, *z* = −4.15, *p* = 0.000, and year-end tests, *z* = −6.30, *p* = 0.000, than in the pretest. Pretest had an average rank of 113.49, post-test average rank was 179.59, 3-month average rank was 178.48, and year-end test had an average rank of 208.00 (Table 5).

## 4. Discussion

The findings supported each hypothesis and mirror the results of [9]. This preliminary study aligns with existing data that corroborates the need for training to prepare frontline mental healthcare responders such as K-12 educators [6,20,21]. Participants’ perceived knowledge of YSP content increased following the intervention. Their perceived knowledge of the actions to take when a student is at risk of suicide and of the signs and symptoms of suicide risk in students both rendered a medium effect. While these gains are important, we would hope to see larger effects from training on participants’ perceptions.

Participants’ confidence increased after YSP intervention training. Their confidence in approaching a student at risk of suicide had a large effect size, signifying perception changes that were observable to the naked eye. Other confidence questions—confidence in helping a student at risk of suicide and asking a student about suicide—produced medium effects. While these effect sizes bode well for the intervention, large effects were desired. We speculate participants’ confidence levels for helping at-risk students and/or asking them about suicide were already elevated before the YSP intervention training.

Participants’ confidence in approaching other adults about students at risk of suicide increased after the intervention. Participants’ confidence in approaching a school counselor about a student at risk of suicide produced a large effect size, indicating that the intervention provided practice and instilled confidence in communicating with other adults. Participants’ confidence in approaching an administrator about a student at risk of suicide produced a medium effect size. The medium effect is a positive indicator about the effectiveness of the YSP intervention, but additional training may be needed. The overall medium and large positive impacts demonstrate that skill-building among school personnel through experiential learning techniques can provide positive outcomes from the YSP intervention training. From our results, we believe school personnel are better equipped to help at-risk youth.

## 5. Limitations

This preliminary study has limitations. While a proportional random sample was used, it was drawn from the total number of training attendees. Not all training attendees answered all questions, including demographic questions, on all tests. The number of participants providing pre-, post-, 3-month, and year-end data is a potential source of bias because of unequal numbers and that some representative population may have been missed due to the stratified random sample and the missing demographic information. We recognized those responding to multiple tests may not have participated in every test, and those not participating could have differed in their perceived knowledge of YSP content or confidence in approaching others about students at risk of suicide. While we respect participants’ rights of refusal to answer any or all survey questions, we believe that more accurate insights on the effects on the YSP training would be drawn from a truly random sample of K-12 school personnel. Likewise, we think other samples from schools that experienced recent traumatic events such as mass school shootings may produce better understanding of the effects of the YSP intervention training.

The knowledge measurements measured perceived rather than true knowledge about youth suicide. This prevents true knowledge gains from being discerned. Differences in test construction and administration could have affected the response set. In future iterations, true knowledge questions and standardized test administration should be used. Validation of the measurement items, scales, and conceptual dimensions may be useful in future studies, especially if researchers wish to establish construct validity beyond a program evaluation outcome. The SARPPD has its own limitations, such as participants’ imperfect memory recall, social desirability, acquiescence, and cognitive dissonance that might cause questionable impact of the true effectiveness of professional development programs. Caution is warranted in extending our results beyond the participants.

Finally, due to the lack of a control group there is limited generalizability of the data, as noted by [33]. MHAGH aims to conduct experimental trials of YSP and other curricula to measure the effectiveness of the Emotional Backpack Program. Adding a control group, such as school staff not in the YSP intervention training or including school personnel from schools without the EBP training, would increase the rigor of these studies.

## 6. Conclusions

K-12 educators provide care for students beyond the classroom, meaning they support students’ personal wellbeing including mental health stability [6]. Being adept with mental health signs and symptoms equips educators with the essential tools to help students navigate and manage aspects of their wellbeing [25]. This preliminary study reinforces the value of training educators to acquire content knowledge and confidence boosting opportunities for engagement in difficult dialogue about suicidality.

The preliminary study revealed that exposure and opportunities for roleplaying to learn how to engage with students experiencing mental health concerns impacts an educator’s preparedness and confidence in such complex situations. Existing research emphasizes increased mental health complexities among K-12 students [2,3], alluding to the demands and intricacies placed on K-12 educators’ roles in and out of the classroom. MHAGH can use these findings to effectively recruit other schools to participate in YSP intervention training, thereby expanding the number of K-12 educators who are prepared to help at-risk youth.

Further, the literature encourages environments that promote transparency about mental health awareness [14]. YSP training helped improve educators’ confidence to engage with others about students’ mental health concerns, calling attention to the importance of identifying early warning signs that may aid in early support and prevention of youth mental health concerns. Texas school districts must provide suicide prevention training annually for new school district and open-enrollment charter school educators [36]. We recommend all Texas school districts use this preliminary study to plan and administer YSP intervention training annually for all school personnel [37].

## Figures and Tables

**Table 1:** Frequencies for nominal and ordinal independent and dependent variables (*n* = 400).

Variables	Categories	*f*	%
Tests	Pretest	175	43.8
	Post-test	116	29.0
	3-month	50	12.5
	Year-end	59	14.8
Race/Ethnicity ^a^	White	98	24.5
	Hispanic	40	10.0
	Black	37	9.3
Gender	Female	164	41.0
	Male	19	4.8
Titles	Teacher	186	46.5
	Another staff ^b^	68	17.0

*Note*. Frequencies may not equal 100% because of missing or unusable data. ^a^ Included American Indian, Asian, and two or more races/ethnicities. ^b^ Included administrator, counselor, paraprofessional, and other titles.

**Table 2:** Tests of normality.

	Kolmogorov– Smirnov ^a^			Shapiro–Wilk		
Statements	*W*	df	*p*	*W*	df	*p*
Knowledge of actions to take	0.20	322	<0.001	0.897	322	<0.001
Knowledge of the signs and symptoms	0.21	322	<0.001	0.896	322	<0.001
Confidence in approaching a student at risk	0.22	322	<0.001	0.901	322	<0.001
Confidence in helping a student at risk of suicide	0.19	322	<0.001	0.907	322	<0.001
Confidence in asking a student about suicide	0.18	322	<0.001	0.912	322	<0.001
Confidence in approaching a school counselor	0.21	322	<0.001	0.867	322	<0.001
Confidence in approaching an administrator	0.22	322	<0.001	0.874	322	<0.001
Confidence in approaching a parent or guardian	0.18	322	<0.001	0.910	322	<0.001

^a^ Lilliefors Significance Correction.

**Table 3:** Descriptive statistics and pairwise comparisons of perceived knowledge of YSP content.

Statements			Tests	*n*	*M* ^a^	*SD*
Knowledge of the actions to take when a student is at risk of suicide.			Pre-	174	3.25	1.03
			Post-	116	3.81	0.82
			3-month	50	3.54	0.81
			Year-end	59	3.95	0.84
			Total	399	3.55	0.96
Knowledge of the signs and symptoms of suicide risk in students.			Pre-	174	3.22	1.01
			Post-	116	3.79	0.79
			3-month	50	3.52	0.84
			Year-end	59	3.98	0.8
			Total	399	3.54	0.94
Pairwise comparisons of knowledge of actions to take when a student is at risk of suicide.						
Sample 1-Sample 2 ^b^	*H*	*SE*	Std. *H*	*p*	Adj. *p* ^c^	
Before-3-Month	−25.911	17.633	−1.469	0.142	0.85	
Before-After	−61.603	13.172	−4.677	<0.001	0.000 *	
Before-Year end	−79.218	16.555	−4.785	<0.001	0.000 *	
3-Month-After	35.693	18.591	1.92	0.055	0.329	
3-Month-Year end	−53.307	21.123	−2.524	0.012	0.07	
After-Year end	−17.615	17.572	−1.002	0.316	1	
Pairwise comparisons of knowledge of the signs and symptoms of suicide risk in students.						
Before-3-Month	−29.899	17.585	−1.7	0.089	0.534	
Before-After	−64.598	13.136	−4.918	<0.001	0.000 *	
Before-Year end	−88.335	16.51	−5.35	<0.001	0.000 *	
3-Month-After	34.699	18.54	1.872	0.061	0.368	
3-Month-Year end	−58.436	21.066	−2.774	0.006	0.033 *	
After-Year end	−23.737	17.524	−1.355	0.176	1	
Before-3-Month	−29.899	17.585	−1.7	0.089	0.534	

*Note.* Before (i.e., pretest) and After (i.e., post-test) relates to participants’ perceptions concerning the training period. ^a^ Measured on a 5-point Likert scale with 1 = poor, 2 = fair, 3 = good, 4 = very good, 5 = excellent. ^b^ Each row tests the null hypothesis that the Sample 1 and Sample 2 distributions are the same. Asymptotic significances (2-sided tests) are displayed. The significance level is 0.05. ^c^ Significance values have been adjusted by the Bonferroni correction for multiple tests. * *p* < 0.05.

**Table 4:** Descriptive statistics and pairwise comparisons of confidence in helping students at risk of suicide.

Statements			Tests	*n*	*M* ^a^	*SD*
Confidence in approaching a student at risk of suicide.			Pre-	175	3.01	1.08
			Post-	116	3.67	0.81
			3-month	50	3.54	0.79
			Year-end	59	4.02	0.80
			Total	400	3.42	1.01
Confidence in helping a student at risk of suicide.			Pre-	173	3.02	1.15
			Post-	116	3.57	0.86
			3-month	49	3.45	0.79
			Year-end	59	3.97	0.81
			Total	397	3.37	1.04
Confidence in asking a student about suicide.			Pre-	175	2.83	1.17
			Post-	115	3.52	0.90
			3-month	50	3.38	0.90
			Year-end	59	3.80	0.91
			Total	399	3.24	1.09
Pairwise comparisons of confidence in approaching a student at risk of suicide.						
Sample 1-Sample 2 ^b^	*H*	*SE*	Std. *H*	*p*	Adj. *p* ^c^	
Before-3-Month	−53.564	17.761	−3.016	0.003	0.015	
Before-After	−70.019	13.261	−5.280	<0.001	0.000 *	
Before-Year end	−111.548	16.674	−6.690	<0.001	0.000 *	
3-Month-After	16.455	18.738	0.878	0.380	1.000	
3-Month-Year end	−57.983	21.290	−2.723	0.006	0.039 *	
After-Year end	−41.529	17.711	−2.345	0.019	0.114	
Pairwise comparisons of confidence in helping a student at risk of suicide.						
Before-3-Month	−39.191	17.798	−2.202	0.028	0.166	
Before-After	−55.998	13.198	−4.243	<0.001	0.000 *	
Before-Year end	−100.330	16.581	−6.051	<0.001	0.000 *	
3-Month-After	16.806	18.738	0.897	0.370	1.000	
3-Month-Year end	−61.139	21.257	−2.876	0.004	0.024 *	
After-Year end	−44.332	17.587	−2.521	0.012	0.070	
Pairwise comparisons of confidence in asking a student about suicide.						
Before-3-Month	−53.264	17.848	−2.984	0.003	0.017 *	
Before-After	−69.290	13.361	−5.186	<0.001	0.000 *	
Before-Year end	−99.065	16.756	−5.912	<0.001	0.000 *	
3-Month-After	16.026	18.855	0.850	0.395	1.000	
3-Month-Year end	−45.801	21.395	−2.141	0.032	0.194	
After-Year end	−29.776	17.824	−1.671	0.095	0.569	

*Note.* Before (i.e., pretest) and After (i.e., post-test) relates to participants’ perceptions concerning the training period. ^a^ Measured on a 5-point Likert scale with 1 = poor, 2 = fair, 3 = good, 4 = very good, 5 = excellent. ^b^ Each row tests the null hypothesis that the Sample 1 and Sample 2 distributions are the same. Asymptotic significances (2-sided tests) are displayed. The significance level is 0.05. ^c^ Significance values have been adjusted by the Bonferroni correction for multiple tests. * *p* < 0.05.

**Table 5:** Descriptive statistics and pairwise comparisons of confidence in approaching other adults about students at risk of suicide.

Statements			Tests	*n*	*M* ^a^	*SD*
Confidence in approaching a school counselor about a student at risk of suicide.			Pre-	103	3.34	1.1
			Post-	116	3.98	0.88
			3-month	50	4.02	0.8
			Year-end	59	4.34	0.76
			Total	328	3.85	0.99
Confidence in approaching an administrator about a student at risk of suicide.			Pre-	103	3.31	1.13
			Post-	116	3.88	0.89
			3-month	50	3.9	0.84
			Year-end	59	4.19	0.82
			Total	328	3.76	1
Confidence in approaching a parent or guardian about a student at risk of suicide.			Pre-	103	2.65	1.08
			Post-	116	3.43	0.93
			3-month	50	3.42	0.97
			Year-end	57	3.74	0.88
			Total	326	3.24	1.06
Pairwise comparisons of confidence in approaching a school counselor about a student at risk of suicide.						
Sample 1-Sample 2 ^b^	*H*	*SE*	Std. *H*	*p*	Adj. *p* ^c^	
Before-3-Month	−52.925	12.264	−4.316	<0.001	0.000 *	
Before-After	−55.294	15.613	−3.542	<0.001	0.002 *	
Before-Year end	−88.27	14.79	−5.968	<0.001	0.000 *	
3-Month-After	−2.369	15.325	−0.155	0.877	1	
3-Month-Year end	−35.345	14.485	−2.44	0.015	0.088	
After-Year end	−32.976	17.412	−1.894	0.058	0.349	
Pairwise comparisons of confidence in approaching an administrator about a student at risk of suicide.						
Before-3-Month	−45.275	12.259	−3.693	<0.001	0.001 *	
Before-After	−46.601	15.607	−2.986	0.003	0.017 *	
Before-Year end	−76.081	14.784	−5.146	<0.001	0.000 *	
3-Month-After	−1.326	15.319	0-.087	0.931	1	
3-Month-Year end	−30.806	14.479	−2.128	0.033	0.2	
After-Year end	−29.48	17.405	−1.694	0.09	0.542	
Pairwise comparisons of confidence in approaching a parent or guardian about a student at risk of suicide.						
Before-3-Month	−64.995	15.655	−4.152	<0.001	0.000 *	
Before-After	−66.101	12.297	−5.376	<0.001	0.000 *	
Before-Year end	−94.515	14.994	−6.304	<0.001	0.000 *	
3-Month-After	1.106	15.366	0.072	0.943	1	
3-Month-Year end	−29.52	17.599	−1.677	0.093	0.561	
After-Year end	−28.414	14.691	−1.934	0.053	0.319	

*Note.* Before (i.e., pretest) and After (i.e., post-test) relates to participants’ perceptions concerning the training period. ^a^ Measured on a 5-point Likert scale with 1 = poor, 2 = fair, 3 = good, 4 = very good, 5 = excellent. ^b^ Each row tests the null hypothesis that the Sample 1 and Sample 2 distributions are the same. Asymptotic significances (2-sided tests) are displayed. The significance level is 0.05. ^c^ Significance values have been adjusted by the Bonferroni correction for multiple tests. * *p* < 0.05.

## Data Availability

Not applicable.
